# Aberrant Upregulation of 14-3-3σ and EZH2 Expression Serves as an Inferior Prognostic Biomarker for Hepatocellular Carcinoma

**DOI:** 10.1371/journal.pone.0107251

**Published:** 2014-09-16

**Authors:** Yi Zhang, Yang Li, Changwei Lin, Jie Ding, Guoqing Liao, Bo Tang

**Affiliations:** 1 Department of Gastrointestinal Surgery, Xiangya Hospital, Central South University, Changsha, P.R. China; 2 Department of Gastrointestinal Surgery, Third Xiangya Hospital, Central South University, Changsha, P.R. China; 3 Department of Gastrointestinal Surgery, Guizhou Provincial People's Hospital, Guiyang, P.R. China; 4 Department of Hepatobiliary Surgery, Affiliated Hospital of Guilin Medical University, Guilin, P.R. China; University of Modena & Reggio Emilia, Italy

## Abstract

Hepatocellular carcinoma (HCC) is the fifth most common malignancy in the world. It is of important significance to find biomarkers for the prognostic monitoring of HCC. The 14-3-3σ and EZH2 proteins are involved in cell cycle regulation and epigenetic silencing. We herein examined the significance of 14-3-3 σ and EZH2 in HCC (n = 167) by immunohistochemistry, RT-PCR and qRT-PCR. The correlation between 14-3-3σ and EZH2 expression and patients' clinicopathologic features were examined, as was the correlation between 14-3-3σ and EZH2 expression and the prognosis of HCC patients. We found that 14-3-3σ and EZH2 were highly expressed in HCC (71% and 90%), the expression of EZH2, but not 14-3-3σ, is associated with vascular invasion and tumor differentiation (*p*<0.01). The coexistence of 14-3-3σ and EZH2 overexpression is associated with a relatively unfavorable prognosis (*p*<0.01), suggesting that aberrant upregulation of 14-3-3σ and EZH2 expression serves as an inferior prognostic biomarker for HCC.

## Introduction

Hepatocellular carcinoma (HCC) is the fifth most common malignancy in the world, and the estimated number of HCC-related deaths exceeds 500,000 per year [1]. The genetic mechanism behind HCC formation is unclear, but it can be concluded that genetic alterations may include cyclins, p27, and p21. However, the functions of some of the associated genes have not been proven, so it is of significant importance for the diagnosis and treatment of liver cancer to explore and study the relationship between associated genes and the generation and development of liver cancer.

The 14-3-3σ protein, which belongs to the 14-3-3 protein family, was originally characterized as an epithelial-specific marker, HME1, which was shown to be responsible for G2 cell cycle checkpoint control by p53 in response to DNA damage in human cells [2]. Moreover 14-3-3σ has been defined as a new class of Cdk inhibitor, as it can bind Cdk2, Cdc2 and Cdk4 and sequester them in the cytoplasm through altered nuclear exporting activities [3]. The 14-3-3σ protein plays important roles in a wide range of regulatory processes, such as mitogenic signal transduction, cell cycle control, and apoptotic cell death [4]. Up- or down-regulation of 14-3-3σ has been found in human cancers. For example, 14-3-3σ expression is lost in breast cancer cells due to promoter hypermethylation [5]. The loss of 14-3-3σ expression was also found in partial HCC tissue, and a significant correlation was found between methylation and loss of expression [6].

Enhancer of zeste homolog 2 (EZH2) is a member of the Polycomb Group (PcG) of proteins. The gene maps to chromosome 7q35 and contains 20 exons and 19 introns [7]. EZH2, the catalytic subunit of PRC2, has a SET domain, which is a typical structural signature of histone methyltransferase activity [8], and gives rise to the methylated version of lysine residue 27 within histone H3 [9]. Moreover, EZH2 is capable of exhibiting DNA methyltransferase activity and can repress the activities of certain genes by gene methylation [10]. Studies have found that the expression of the EZH2 gene in cancer was significantly higher than in paraneoplastic or normal tissue [11]. Silencing EZH2 expression in liver cancer cells attenuated liver cancer proliferation and metastasis [12].

The expression of 14-3-3σ is determined by the methylation status of the gene, and EZH2 is also a methylation-regulated gene. Is there a correlation between 14-3-3σ and EZH2 expression? What type of relationship exists between the gene expression and the clinicopathological features and prognosis of HCC? Our study further investigates the above issues. We determined that there is no correlation between 14-3-3σ and EZH2 expression and that EZH2 is associated with tumor differentiation and vascular infiltration. In addition, the combination of 14-3-3σ and EZH2 can predict the prognosis of HCC, which suggests that no regulatory relationship exists between 14-3-3σ and EZH2 expression, but the combination of the two genes could become candidate indicators for monitoring the prognosis of HCC.

## Materials and Methods

### Patient samples

The study was reviewed and approved by ethics committee of affiliated hospital of Guilin medical university and written informed consent was obtained from all patients. The study included 167 patients with HCC aged from 25 to 74 years; all patients underwent curative surgery from 2006 to 2008 at the Department of Hepatobiliary Surgery, The Affiliated Hospital of Guilin Medical University. No patients underwent palliative resection, preoperative chemotherapy, or radiotherapy. Clinicopathological features examined included age, gender, etiology, presence of liver cirrhosis, AFP, tumor size, tumor differentiation, vascular invasion, and tumor stage. Tumors were classified and graded based on the pTNM classification advocated by the International Union Against Cancer. All 167 patients were followed for 5 years with computed tomography and ultrasonography every six months after discharge.

### Immunohistochemistry

Specimens were fixed with 10% formaldehyde, embedded in paraffin, and sectioned into 4-µm-thick slices. Sections were deparaffinized by prolonged incubation in xylene (3–4 min), followed by prolonged washing and rehydration in ethanol (96% ethanol for 2–3 min, 80% ethanol for 3 min, and 70% ethanol for 3 min). After deparaffinization, endogenous peroxidase was blocked by a 0.3% hydrogen peroxidase-methanol solution for 30 minutes. For antigen retrieval, sections were pretreated with citrate buffer for 15 minutes at 100°C in a microwave oven. After blocking with phosphate-buffered saline (PBS) plus 3% skim milk at room temperature for 2 h, the blocked sections were incubated overnight at 4°C with primary antibody for 14-3-3σ (sc-7681, Santa Cruz, USA) or EZH2 (ab189201, abcam, USA) at a dilution of 1 : 100. After washing, the sections were reacted with biotinylated rabbit anti-goat IgG (Santa Cruz, USA) or goat anti-rabbit IgG (Santa Cruz, USA) at a dilution of 1 : 500, followed by incubation with an avidin–biotin peroxidase complex. The immune complex was visualized with diaminobenzidine as the substrate. The sections were rinsed briefly in water, counterstained with hematoxylin, and mounted. In addition, sections incubated without the primary antibody were used as the negative controls.

If the HCC accompany with liver fibrosis. The METAVIR scoring system was used to stage liver fibrosis as follows: F1, portal fibrosis without septa; F2, portal fibrosis and few septa; F3, numerous septa without cirrhosis; F4, cirrhosis [13]. Scores of F3 or F4 were considered to indicate advanced fibrosis.

Two investigators independently evaluated the immunohistochemical staining. Ten high power fields (×200) were randomly selected for quantification. The percentage of 14-3-3σ or EZH2 positive tumor cells were estimated as follows: 0 point, <1%; 1 point, 1–25%; 2 points, 26–50%; 3 points, 51–75%; 4 points, >75%. The score of the staining intensity was presented as follows: 0 point, no staining; 1 point, weak staining; 2 point, moderate staining; 3 point, strong staining. Then, the two scores were multiplied to obtain a combination score ranging from 0 to 12, with 0 representing no staining (-), 1–3 points representing weak intensity (+); 4–6 points representing moderate intensity (++); 8–12 points representing strong intensity (+++). Protein expression was defined as high when the combination scores were >3 and low when combination scores were≤3.

### Western blot

Frozen tissues were homogenized and lysed with lysis buffer (50 mM Tris–HCl, 137 mM NaCl, 10% glycerol, 100 mM sodium orthovanadate, 1 mM phenylmethylsulphonyl fluoride (PMSF), 10 mg/ml aprotinin, 10 mg/ml leupeptin, 1% Nonidet P-40, 5 mM protease inhibitor cocktail; pH 7.4). After the determination of the protein concentration using BCA kit assay, β-mercaptoethanol and bromophenol blue were added to the sample buffer for electrophoresis. Proteins was separated by 10% PAGE and transferred to polyvinylidene difluoride membranes (Bio-Rad, USA). The membranes were incubated with primary antibody overnight at 4 °C. After incubation with secondary antibody for another 2 h, reactive bands were visualized using the enhanced chemiluminescence system. The band intensity was quantified using an image analysis system (Quantity One v4.62).

### RNA extraction and reverse transcriptase PCR (RT-PCR)

Total RNA from tissue was prepared using RNAisoTM Plus (Takara, Japan) according to the manufacturer's instructions. The concentration of the total RNA samples was determined with a spectrophotometer (Beckman Coulter, USA). The primers specific for 14-3-3σ and EZH2 were synthesized by Invitrogen Biotechnology Co. Ltd., China. The primers for amplification were as follows: Human 14-3-3σ forward (5’-AGAAGCGCATCATTGACTCA-3’), reverse (5’-CTGTTGGCGATCTCGTAGTG-3’); EZH2 forward (5’-GCCAGACTGGGAAGAAATCTG-3’), reverse (5’-TGTGCTGGAAAATCCAAGTCA-3’). The RT-PCR was performed using an RT-PCR kit (TaKaRa, Japan) according to the manufacturer's instructions. PCR was performed with the following conditions: initial denaturation at 94 °C for 2 min, then 35 cycles at 95°C for 30 s, 55 °C for 45 s and 72 °C for 70 s, and a final extension at 72 °C for 5 min. The PCR products were separated on 1.5% agarose gels by electrophoresis and visualized with UV light.

### Quantitative real time RT-PCR (qRT-PCR)

Gene expression was evaluated again by the quantitative real-time RT-PCR method. Total RNA was prepared from HCC specimens using an RNeasy Mini Plus Kit (Qiagen, Holland) and the quality was evaluated using an Agilent 2100 Bioanalyzer (Agilent Technologies, USA) as described above. One microgram of total RNA per 20 µl of reaction mixture was converted to cDNA using a High Capacity cDNA Reverse Transcription Kit (Life Technologies, USA). Quantitative real-time PCR was performed with SYBR Premix Ex Taq (Takara, Japan) on a GeneAmpV R 7300 Sequence Detection System (Life Technologies, USA) in accordance with the manufacturer’s protocol. The primers for amplification were as follows: Human 14-3-3σ forward (5’-AGAAGCGCATCATTGACTCA-3’), reverse (5’-CTGTTGGCGATCTCGTAGTG-3’); EZH2 forward (5’-GCCAGACTGGGAAGAAATCTG-3’), reverse (5’-TGTGCTGGAAAATCCAAGTCA-3’).

### Statistical analysis

Statistical analysis was performed using SPSS 17.0 software (SPSS Inc., USA). χ^2^ tests were used to evaluate the relationship between the expression and clinicopathological variables. Spearman’s correlation coefficient was used to calculate correlations between 14-3-3σ and EZH2. The Kaplan-Meier analysis was employed for survival analysis, and differences in survival probabilities were estimated using the log-rank test. The Cox proportional hazards model was used to determine the independent factors of survival. *p*<0.05 was considered statistically significant.

## Results

### 14-3-3σ and EZH2 expression in normal tissue, fibrotic tissue and liver cancer

**Figure 1:**
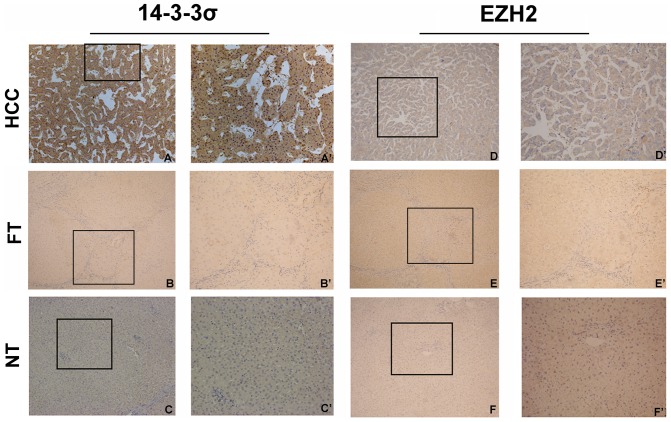
Immunohistochemical staining for 14-3-3σ and EZH2 in hepatocellular carcinoma, fibrotic tissues and normal adjacent tissues. (A) 14-3-3σ was strong stained in hepatocellular carcinoma tissue, (B) nearly negative expression of 14-3-3σ in fibrotic tissue, (C) with an almost negative expression level in paired normal adjacent tissue (100×); (D) EZH2 was overexpressed in the cytoplasm in hepatocellular carcinoma tissue, (E) almost negative expression of EZH2 in fibrotic tissue, (F) nearly absent in paired normal tissues from the same case (100×). (A’, B’ and C’) and (D’, E’ and F’) demonstrated the higher magnification (200×) from the area of the box in (A, B and C) and (D, E and F), respectively.

**Figure 2:**
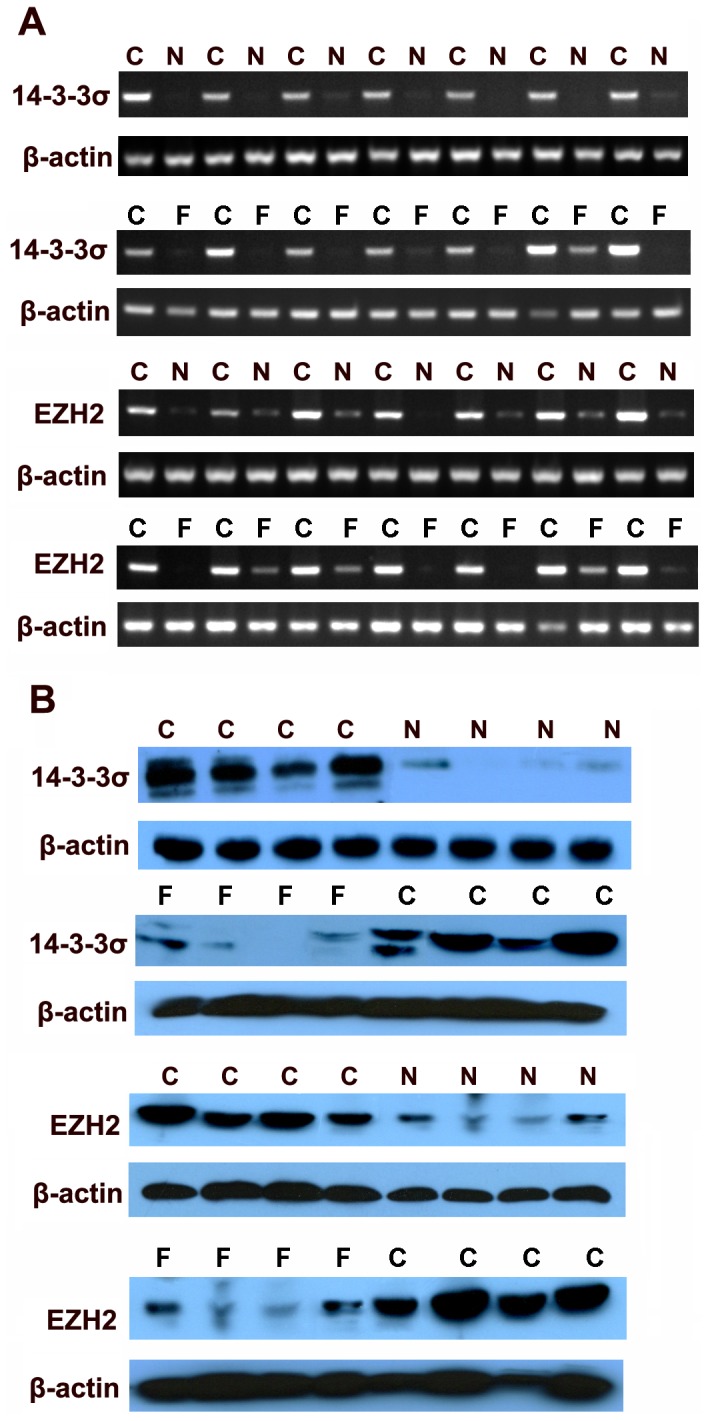
The expression of 14-3-3σ and EZH2 in hepatocellular carcinoma, fibrotic tissues and normal adjacent tissues as determined by RT-PCR and Western blot. (A) The expression of 14-3-3σ and EZH2 was detected by RT-PCR, C: Cancer tissue, N: Noncancerous tissue, F: fibrotic tissues. (B) The immunoblot analysis of 14-3-3σ and EZH2. β-actin was used as endogenous reference.

**Figure 3:**
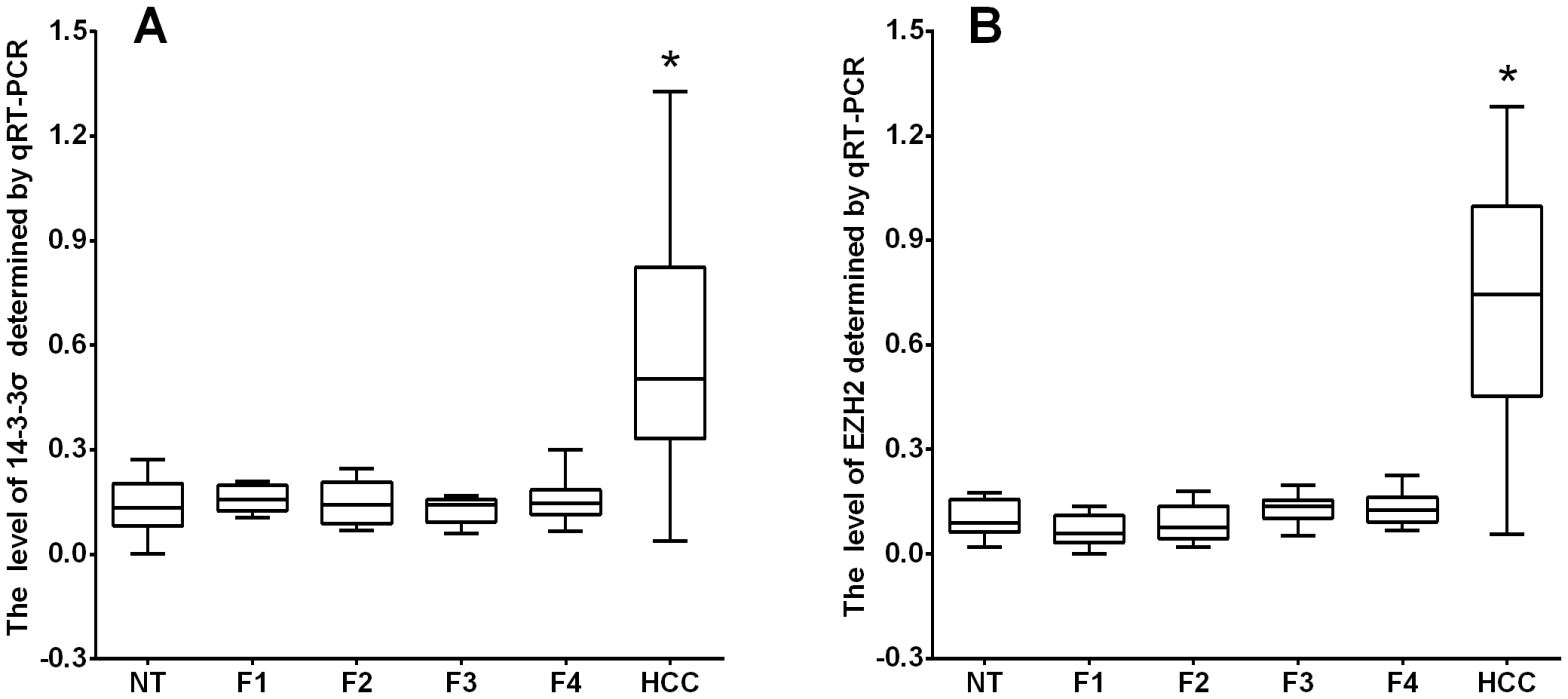
Expression levels of 14-3-3σ and EZH2 quantitatively determined by real-time RT–PCR. (A) Expression levels of 14-3-3σ in hepatocellular carcinoma, fibrotic tissues and normal adjacent tissues, liver fibrosis was classified into four stages, F1 to F4, according to METAVIR scoring system. (B) Expression levels of EZH2 quantitatively in hepatocellular carcinoma, fibrotic tissues and normal adjacent tissues. The correction values were calculated by dividing the 14-3-3σ and EZH2 amounts by the amount of β-actin concurrently examined on the same samples (*, *p*<0.05).

Many HCC often arise within the background of liver fibrosis, so we detected the expression of 14-3-3σ and EZH2 in liver cancer with normal tissue and liver fibrotic tissue as control by RT-PCR, qRT-PCR, Western blot and immunohistochemistry. 14-3-3σ was detected in the cytoplasm of cells in 71% (119/167) of HCC patients, whereas adjacent normal tissue and liver fibrosis tissues showed negative expression. On the contrary, EZH2 immunostaining was detected in a nuclear staining pattern. Out of all HCC tissues, 90% (150/167) were immunopositive for EZH2, whereas the adjacent normal tissues and liver cirrhosis tissues were immunonegative or weak for EZH2 (Figure 1). The results of RT-PCR and Western blot also revealed similar findings (Figure 2). Furthermore, we divided liver fibrosis into four stages according to METAVIR scoring system, and detect the expression of 14-3-3σ and EZH2 in different stage of liver fibrosis (n = 110), normal tissue (n = 57) and liver cancer by qRT-PCR(Figure 3). The results indicated that there was no significant difference in 14-3-3σ expression between different stages F1 to F4, 14-3-3σ expression in HCC was significantly higher than other group. Interestingly, in EZH2 expression group, we found an increase trend from F1 to F4 stage, but no significant difference existed between each stage and normal tissue.

**Figure 4:**
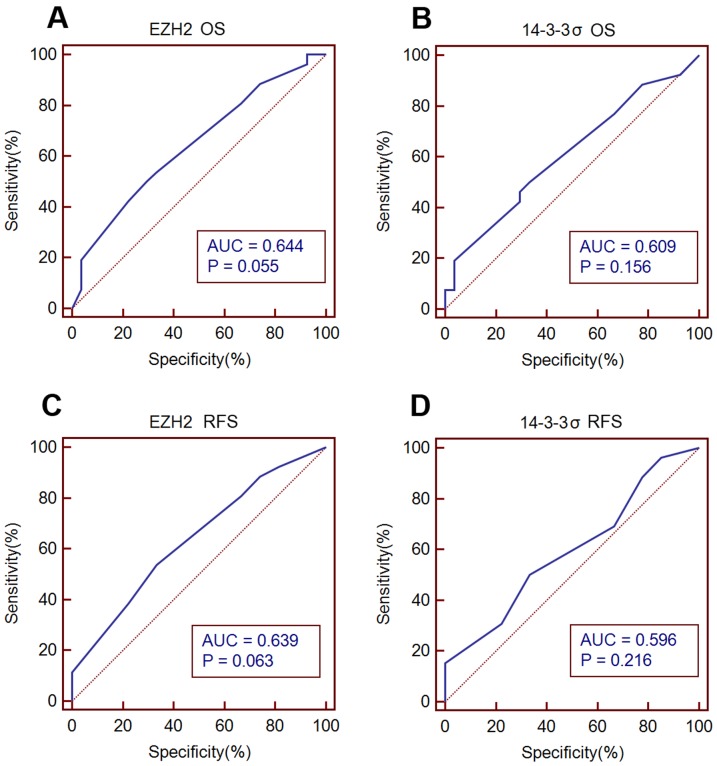
ROC curve analysis to determine cutoff score for 14-3-3σ and EZH2 expression. (A, C) EZH2 cutoff point of OS and DFS, the EZH2 cutoff score for OS and DFS was 4 (*p* = 0.055) and 4 (*p* = 0.063). (B, D) 14-3-3σ cutoff point for OS and DFS. The 14-3-3σ cutoff scores for OS and DFS were 3 (*p* = 0.156) and 3 (*p* = 0.216), respectively. At each immunohistochemical score, the sensitivity and specificity for the outcome being studied was plotted, thus generating a ROC curve.

To further assess the survival analysis and to avoid the problems of multiple cutpoint selection, ROC curve analysis was employed to determine the cutoff score for 14-3-3σ and EZH2 expression. As shown in Figure 4, the 14-3-3σ cutoff scores for OS and DFS were 3 (*p* = 0.156) and 3 (*p* = 0.216), respectively; the EZH2 cutoff scores for OS and DFS were 4 (*p* = 0.055) and 4 (*p* = 0.063). We thus selected a 14-3-3σ expression score of 3 (> 3 VS ≤3) and EZH2 expression score of 4 (>4 VS≤4) as the uniform cutoff point for survival analysis (Figure 3).

### 14-3-3σ and EZH2 expression and clinicopathologic features

The association of 14-3-3σ and EZH2 expression with pathological variables was examined. 14-3-3σ expression in HCC was not statistically associated with clinicopathologic features. On the other hand, the incidence of vascular invasion and poor tumor differentiation was higher in the high EZH2 group than in the EZH2 low group. No significant differences in host factors, such as the patient's age, gender, tumor stage, tumor size, etc. were observed between the high and low EZH2 groups (Table 1).

**Table 1:** Relationship between 14-3-3σ and EZH2 and clinicopathological parameters in 167 HCC patients.

Variables	All cases	14-3-3σ expression		*χ^2^*	*P* *	EZH2 expression		*χ^2^*	*P* *
		High	Low			high	low		
**Age(years)**									
≥50	93	59	34			70	23		
<50	74	39	35	0.041	0.161	57	17	1.581	0.791
**Gendar**									
Male	120	70	50			92	28		
Female	47	28	19	2.464	0.883	35	12	1.408	0.765
**Liver cirrhosis**									
Positive	110	68	42			86	24		
negative	57	30	27	0.104	0.252	41	16	0.231	0.369
**Etiology**									
viral	109	66	43			79	30		
Non-viral	58	32	26	0.458	0.502	48	10	0.030	0.138
**Serum AFP(ng/ml)**									
≤200	93	57	36			66	27		
>200	74	41	33	0.345	0.443	61	13	0.011	0.085
**Tumor stage**									
I	30	19	4			23	7		
II	49	28	21			31	18		
III	67	38	29			55	12		
IV	21	13	8	0.694	0.125	18	3	0.484	0.079
**Tumor size(cm)**									
≤5	29	13	16			20	9		
>5	138	85	53	0.014	0.096	107	31	0.176	0.326
**Tumor differentiation**									
Well	34	17	17			25	9		
Moderate	101	57	44			84	17		
Poor	32	24	8	0.192	0.092	18	14	0.015	0.007
**Vascular invasion**									
Yes	72	45	27			61	11		
No	95	53	42	0.250	0.383	66	29	0.000	0.022
14-3-3σ		EZH2				rs			*p*

* *Probability, P, from* *χ^2^ test*.

### Relationship between 14-3-3σ and EZH2 expression

We wondered whether EZH2 could regulate the expression of 14-3-3σ by methylation, so we examined the relationship between 14-3-3σ and EZH2. The results showed that the Spearman's correlation coefficient between 14-3-3σ and EZH2 was -0.054 (*p* = 0.492), suggesting no association between the two parameters (Table 2).

**Table 2:** Correlation analysis between expression of 14-3-3σ and EZH2 in HCC.

14-3-3σ	EZH2	rs	*p*
	+++	++	+	-	Total		
+++	8	15	10	4	37	−0.054	0.492
++	17	35	8	1	61		
+	7	9	2	3	21		
-	21	15	4	8	48		
total	53	74	24	16	167		
	**Overall survival**				**Relapse-free survival**		

### 14-3-3σ and EZH2 expression and survival analysis

**Figure 5:**
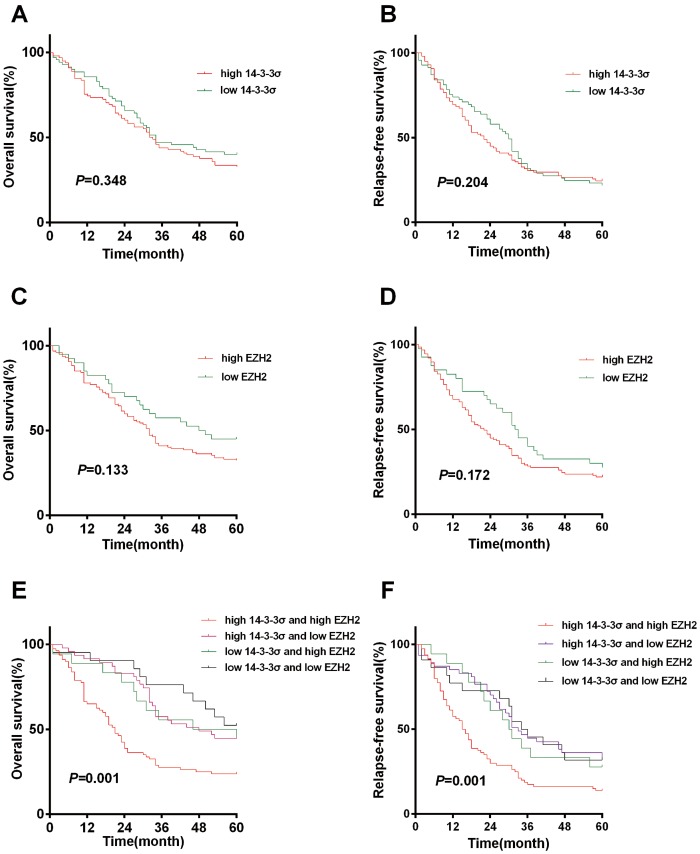
Kaplan-Meier survival curves with regard to disease-free-survival and overall survival according to 14-3-3σ and EZH2 expression. (A) There are no significant differences in OS between patients with positive (32.65%) and negative (40%) staining for 14-3-3σ. (B) There are no significant differences in RFS between patients with positive (24.49%) and negative (21.74%) expressions of 14-3-3σ. (C) There are no significant differences in OS between patients with positive (32.28%) and negative (45%) expressions of EZH2. (D) There are no significant differences in RFS between patients with positive (24.49%) and negative (21.74%) expressions of EZH2. (E) The five-year OS of the coexpressed high 14-3-3σ and EZH2 group were 23.75%, significantly worse than the three other groups (*p*<0.05). (F) The five-year OS of coexpressed high 14-3-3σ and EZH2 group were 23.75%, significantly worse than three other groups (*p*<0.05).

We carried out follow-up for patients out to five years. Kaplan-Meier analysis shows that the five-year OS rates were 32.65% and 40% in the 14-3-3σ positive and negative groups, respectively, and the five-year RFS were 24.49% and 21.74% in both groups. Although the OS rate of the 14-3-3σ negative group was better than the positive group (40% vs. 32.65%). No significant differences were detected between the two groups (Figure 5, *p* = 0.348). Kaplan-Meier analysis also shows that the OS of the EZH2 high and low groups were 32.28% and 45%, respectively, and the RFS of EZH2 was 22.05% and 27.5%. There is no significant difference between the EZH2 high and low groups (Figure 5, *p* = 0.172).

Then, the presence of 14-3-3σ and EZH2 overexpression was investigated to verify their correlation with patients' survival. We found that the five-year OS and RFS of coexistent high 14-3-3σ and EZH2 groups were 23.75% and 13.75%, significantly worse than three other groups (Figure 5, *p* for OS and RFS were 0.001 and 0.001). This difference is statistically significant.

### Multivariate Cox regression analysis

Multivariate analysis showed the following factors to be significantly related to survival: liver cirrhosis, stage, size, and vascular invasion. Multivariate regression analysis indicated that expression of EZH2 and expression of 14-3-3σ were not independent prognostic factors (Table 3).

**Table 3:** Multivariate survival analysis of five-year overall and relapse-free survival in 167 patients with hepatocellular carcinoma.

	Overall survival			Relapse-free survival		
variable	Hazard Ratio	95% confidence interval	*P*	Hazard Ratio	95% confidence interval	*P*
Age	0.801	0.437–1.529	0.501	0.865	0.345–2.155	0.748
Gendar	0.937	0.617–1.434	0.752	1.175	0.539–2.601	0.696
Liver cirrhosis	1.558	1.020–2.357	0.035	2.056	1.113–3.766	0.025
etiology	1.554	0.542–4.510	0.424	1.755	0.693–4.434	0.245
AFP	1.241	0.451–2.031	0.490	1.438	0.743–2.743	0.281
Stage	3.358	2.090–5.398	0.001	2.913	1.878–4.698	0.001
size	2.608	1.093–6.211	0.035	2.040	1.062–2.889	0.036
differentiation	1.443	0.969–2.184	0.089	1.454	0.977–2.171	0.074
Vascular invasion	3.505	2.157–5.724	0.000	2.973	1.835–4.825	0.000
14-3-3σ	1.410	0.590–3.353	0.442	1.213	0.604–2.449	0.594
EZH2	1.843	0.645–5.310	0.262	1.993	0.925–4.351	0.082

## Discussion

Several reports have noted that EZH2 was over-expressed in most HCC resection tissues by immunohistochemistry, whereas it was negatively expressed in nearly all of the corresponding non-tumor tissues [14], [15], which was consistent with our findings. We found that the expression rate of EZH2 was 90% in 167 HCC specimens and that the staining intensity was significantly higher than in liver fibrotic tissues and normal tissues. However, the expression level of 14-3-3σ in our study was inconsistent with an investigation by Norikazu Iwata, whose finding showed 5/19 HCC tissues have 14-3-3σ expression [6]. We found that the expression rate of 14-3-3σ was 71%, significantly higher than 5/19. The reason may the differences in the number of samples; a sample size of 19 is too small compared to 167 samples in our study. Our findings also suggested that EZH2 and 14-3-3σ may become hopeful biomarkers to distinguish liver cancer from non-tumor tissues.

Although the association between clinicopathological variables and 14-3-3σ has been well documented for gastric cancer [16], [17] and breast cancer [18], little is known about the association in liver cancer. We examined the clinicopathological features of 14-3-3σ expression in HCC. We observed no statistically significant association between 14-3-3σ expression and clinicopathological features. Increased expression of EZH2 in HCC has been documented in many studies [19]; our study found that EZH2 expression was closely associated with tumor differentiation and vascular infiltration. These findings are compatible with a previous study in which upregulated EZH2 was shown to be associated with tumor progression, especially facilitating portal vein invasion in human HCC [20]. The association of EZH2 with tumor differentiation may be related to the major biological function of EZH2 which is to maintain the undifferentiated stage of cells [21]. Molecular mechanisms linking high EZH2 expression with increased vascular infiltration in HCC has not been well defined, but Au SL et al. reported that EZH2 overexpression can activate Rho/ROCK signaling by inactivating DLC1 to promote liver metastasis [22].

One important role of EZH2 in cancer is the epigenetic repression of tumor suppressor genes by histone modification and promoter methylation. Emmanuelle et al. demonstrated that EZH2 is required for DNA methylation of EZH2-target promoters via interactions with DNA methyltransferases [23]. Additionally, 14-3-3σ is regarded as tumor suppressor gene that is a negative regulator of the cell cycle G2-M phase checkpoint [24]. Norikazu et al. provide evidence that hypermethylation results in the loss of the 14-3-3σ in HCC [11]. Therefore, we examined whether 14-3-3σ expression is regulated by EZH2. We examined the association between 14-3-3σ and EZH2, and no correlation was found between their expression levels, which indicated the methylation of 14-3-3σ might be controlled by factors other than EZH2.

We also investigated the correlation of EZH2 and 14-3-3σ expression with the prognosis of HCC. Although the OS rate in the 14-3-3σ -negative group was better than the positive group, this difference was not statistically significant. This finding appears to be consistent with previous studies [9]. As a possible explanation, EZH2 expression is strongly associated with prognosis only in patients with malignancies from hormonally regulated tissues, such as breast and prostate [25]. The correlation between 14-3-3σ and prognosis has not been reported to date, and we found no significant differences in the OS and RFS rates between the low and high group. Then, the combination of 14-3-3σ and EZH2 was applied to investigate their correlation with prognosis. Interestingly, the coexistence of 14-3-3σ and EZH2 high groups have the worst survival relative to the other three groups. The presence of 14-3-3σ and p53 overexpression may be considered as a significant predictor of OS and RFS in HCC.

In conclusion, we demonstrated in a large study population with HCC that 14-3-3σ and EZH2 are immunopositive in 71% and 90% of the patients correspondingly. Additionally, we found that 14-3-3σ has no correlation with clinicopathological features and that EZH2 was associated with tumor differentiation and vascular infiltration. Moreover, the presence of 14-3-3σ and EZH2 overexpression identifies a population of patients with an unfavorable prognosis, which can be considered a significant predictor of OS and RFS in HCC. However, the precise function of EZH2 and 14-3-3σ in HCC remains unclear, and further investigation is needed to clarify the relationship between EZH2, 14-3-3σ and HCC progression.
